# Ectopic kidney injury due to blunt abdominal trauma

**DOI:** 10.1093/jscr/rjad065

**Published:** 2023-02-23

**Authors:** Vaia Karapepera, Konstantinia Kofina, Nikolaos Papatheodorou, Eleni Effraimidou, Michael Karanikas

**Affiliations:** 1st University Surgical Department, University Hospital of Alexandroupolis, Democritus University of Thrace, Alexandroupolis 68100, Greece; 1st University Surgical Department, University Hospital of Alexandroupolis, Democritus University of Thrace, Alexandroupolis 68100, Greece; 1st University Surgical Department, University Hospital of Alexandroupolis, Democritus University of Thrace, Alexandroupolis 68100, Greece; 1st University Surgical Department, University Hospital of Alexandroupolis, Democritus University of Thrace, Alexandroupolis 68100, Greece; 1st University Surgical Department, University Hospital of Alexandroupolis, Democritus University of Thrace, Alexandroupolis 68100, Greece

## Abstract

Ectopic kidney is a relatively uncommon anatomic variation that is usually detected incidentally in patients undergoing imaging for an unrelated reason. Most cases are asymptomatic and are often revealed by a complication; however, ectopic kidney is generally associated with higher risk of traumatic injury, urinary tract infection, renal calculi and other urologic conditions. We report the case of a 65-year-old male patient with a post-traumatic renal laceration on a previously undiagnosed ectopic pelvic kidney, with successful conservative treatment.

## INTRODUCTION

An ectopic kidney or renal ectopia is a kidney not located in its proper anatomic position due to abnormal migration from the fetal pelvis during fetal development [1]; it can therefore be located anywhere along the path from the pelvis to the upper retroperitoneum. When the kidney remains in the pelvic fossa, it is called a pelvic kidney, as it fails to rise from the pelvis in its metanephros stage.

The incidence of ectopic pelvic kidney is one out of 3000 births; however, only 10% of these cases are diagnosed during their lifetime [2]. Most cases remain asymptomatic, although they are generally associated with higher risk of urinary infection, renal calculi and other conditions. Most importantly, the ectopic kidney is more susceptible to trauma, as the ectopic site seems to be less protected anatomically. Moreover, the pelvic kidney is considered to be slightly more mobile and is thus more prone to trauma, even on a low velocity mechanism.

Herein, we report an interesting case of post-traumatic renal laceration on an ectopic pelvic kidney after blunt abdominal injury.

## CASE REPORT

A 65-year-old male patient was referred to the emergency department of our institution due to blunt right-sided thoracic and abdominal trauma after falling from a 2-m height, complaining about shortness of breath, right-sided chest pain and lower abdominal pain. Initial vital signs included relatively low blood pressure (98/65 mmHg), mild tachycardia (110 beats per minute), a respiratory rate of 16 breaths per minute and oxygen saturation of 95%. His medical history included no concomitant pathologies.

Clinical examination revealed tenderness on the right lower ribs, with slightly diminished respiratory sounds, and lower abdominal tenderness upon palpation. Macroscopic hematuria was present while Foley catheter placement, without indications of iatrogenic trauma. Laboratory exams showed hemoglobin of 13 g/dl and hematocrit (Ht) of 37.4%.

**Figure 1:**
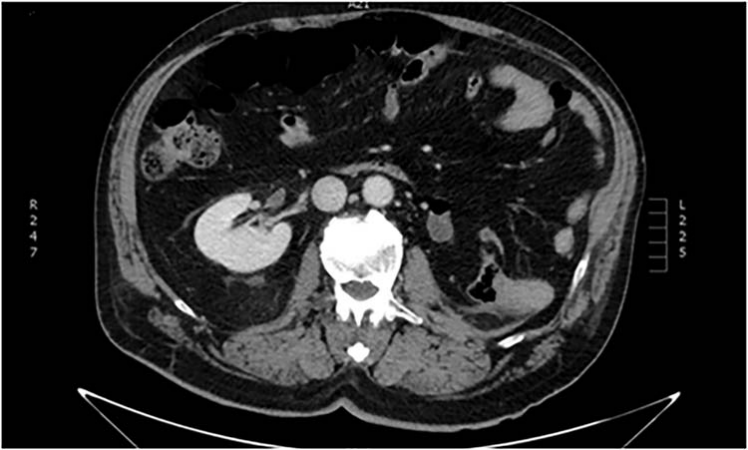
Abdominal CT imaging. Absence of left kidney from its usual anatomic position.

**Figure 2:**
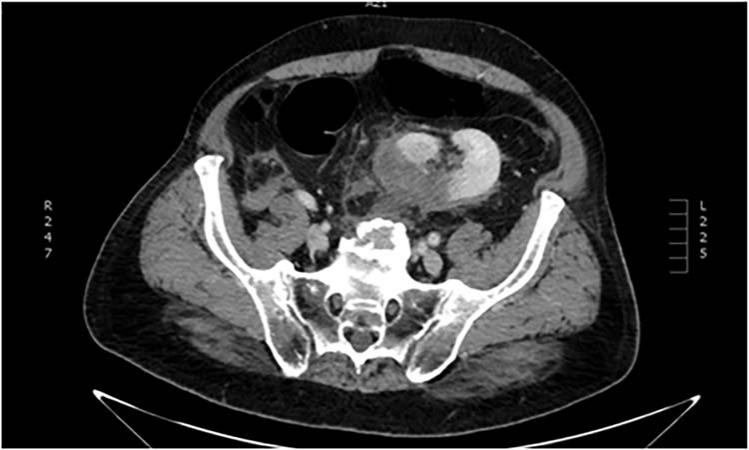
Abdominal CT imaging. Ectopic (pelvic) left kidney. Presence of grade III laceration without collecting system rupture or urinary extravagation.

Imaging investigation with thoracic x-ray revealed 9th to 11th posterior rib fractures, as well as small pneumothorax on the right rib cage. Abdominal ultrasound revealed no internal bleeding or fluid within the peritoneal cavity and spaces, but an injured ectopic left kidney was detected within the pelvic cavity, previously unbeknownst to the patient. Further imaging with abdominal and pelvic computed tomography (CT) scan confirmed the occurrence of a grade III laceration (according to the American Association of the Surgery of Trauma—AAST renal injury grading scale) of the ectopic kidney (Figs. 1 and 2).

The patient was admitted to our surgical department and, after hemodynamic stability was ensured, conservative treatment was decided, after urologic consultation, and in accordance with the AAST guidelines. Thoracic trauma was treated with Büllau tube placement and the renal injury was re-evaluated on the fourth day with a new abdominal CT scan, which indicated no internal bleeding within the peritoneal cavity, no urinary bleeding and significant renal imaging improvement. The patient’s recovery was uneventful and he was discharged on the seventh day in good health condition.

## DISCUSSION

Kidney development begins during the sixth to eighth week of fetal development, when the kidneys ascend from the pelvis towards their normal position between the transverse processes of T12 to L3 vertebrae, with the left kidney typically somewhat more superiorly placed than the right. Failure of ascent of the kidney will cause the kidney to remain in the pelvis, outside the normal renal fossa, leading to the condition of ectopic kidney [3].

**Figure 3:**
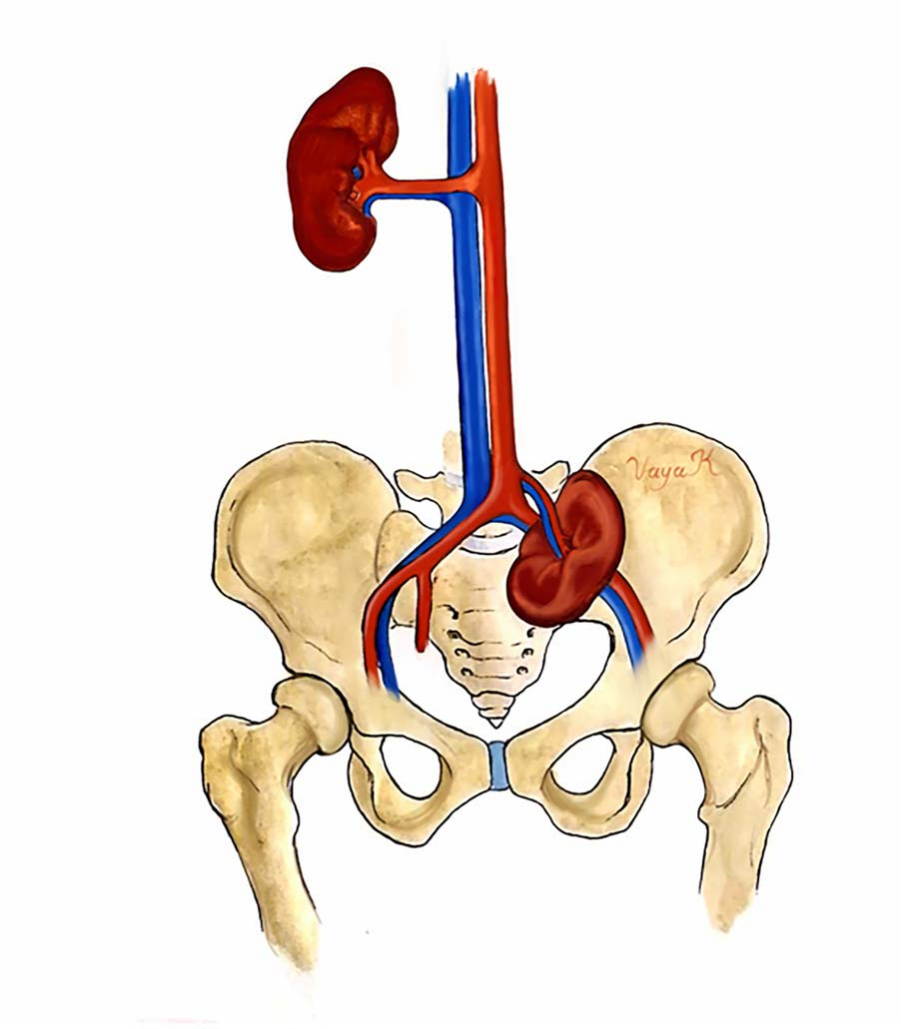
Anatomic illustration. Location of the pelvic kidney below the aortic bifurcation.

The kidney may be ectopic on its own side (simple ectopia) or across the midline and be fused or unfused with the contralateral kidney (crossed ectopia). The final site of the metanephros determines the location of renal ectopia; the most common position is inside the pelvic cavity, just below the aortic bifurcation [4], as in our case (Fig. 3). Vascular supply is not consistent, and the ectopic kidney may receive vascular access from a range of vessels, such as the iliac, mid sacral or hypogastric arteries [5]; understanding this anatomy, thus, is essential for any surgeon operating on a patient with an ectopic kidney.

Because of the rare or vague symptoms, only one in 10 000 patients with ectopic kidney is recognized clinically. Even though no treatment is required in asymptomatic cases, ectopic kidney has been associated with several conditions and urologic complications, such as urinary tract infections and renal calculi. The pelvic kidney can also be correlated with other urogenital anomalies, as well as gastrointestinal, cardiovascular and skeletal disorders [3, 6]. The patient should be advised to have regular follow-up ultrasounds in order to detect complications.

Most importantly, the ectopic kidney is thought to be more susceptible to trauma than the normally positioned kidney. Indeed, ordinary kidney trauma is uncommon, because it is well shielded by the rib cage, spinal and abdominal muscles and vertebral column; perirenal fat and the Gerota fascia also consist an extra protective layer. Lack of these elements make the ectopic kidney far more exposed and susceptible to blunt or penetrating injury [7]. Moreover, the pelvic kidney is thought to be slightly more mobile and thus more susceptible to the shock of a blow.

In most cases, low grade (AAST I, II and III) injury can be managed conservatively [8], as in our case. Non-operative management includes supporting care, bed rest, vital signs and laboratory test monitoring a re-imaging when there is any deterioration, and use of minimally invasive procedures (angioembolization or ureteral stenting) if indicated. However, in Grade IV and V injuries, surgical management is essential. Absolute indications for surgical intervention are hemodynamic instability and unresponsiveness to aggressive resuscitation due to renal hemorrhage, grade 5 vascular injury and an expanding or pulsatile perirenal hematoma found during laparotomy performed for associated injuries [9].

In conclusion, because of its rarity and non-typical symptoms, ectopic kidney diagnosis is often an unsuspected finding. Misdiagnosis of ectopic renal injury may can lead to life-threatening results. Early detection and recognition of the ectopic kidney can prevent complications and enhance recovery. Surgeons must be aware of the associated conditions and complications, so that patients with ectopic kidney can be assessed and treated appropriately.

## CONFLICT OF INTEREST STATEMENT

None declared.

## FUNDING

No funding was received for the preparation and publication of this case report.
